# 11种吸附剂上二噁英的热吸附/脱附性能评价

**DOI:** 10.3724/SP.J.1123.2020.10009

**Published:** 2021-04-08

**Authors:** Lin WANG, Longxing WANG, Yuwen NI, Haijun ZHANG, Jiping CHEN

**Affiliations:** 1.中国科学院大连化学物理研究所, 中国科学院分离分析化学重点实验室, 辽宁 大连 116023; 1. CAS Key Laboratory of Separation Science for Analytical Chemistry, Dalian Institute of Chemical Physics, Chinese Academy of Sciences, Dalian 116023, China; 2.中国科学院大学, 北京 100049; 2. University of Chinese Academy of Sciences, Beijing 100049, China

**Keywords:** 二噁英, 吸附剂, 在线检测, 热吸附, 热脱附, dioxin, adsorbent, online monitoring, thermal adsorption, thermal desorption

## Abstract

筛选在低温下高效捕集并在一定的高温下可以快速完全脱附二噁英的吸附/脱附材料是二噁英在线热捕集的关键。该研究以1,2,3,4-四氯代二苯并-对-二噁英(1,2,3,4-TCDD)和1,2,3,8,9-五氯代二苯并呋喃(1,2,3,8,9-PCDF)为二噁英模型物,以电子捕获检测器(ECD)作为检测器,利用填充柱气相色谱系统,测定了这两种二噁英单体在11种吸附剂上4~5个温度点下的保留体积,并建立了相应的范特霍夫方程。结果表明,11种吸附剂的线性方程决定系数(*R*^2^)均大于0.96。根据范特霍夫方程,预测了吸附剂在120、150、180 ℃时的气固分配系数(*K*_SA_)。弗洛里硅土在120、150、180 ℃ 3个温度点下都具有最强的吸附能力,特别是在120 ℃时,1,2,3,4-TCDD、1,2,3,8,9-PCDF在弗洛里硅土上的*K*_SA_分别高达1.82×10^8^ m^3^/g、1.46×10^13^ m^3^/g。碳基吸附剂的高分子多孔微球GDX系列的GDX-101、GDX-102、GDX-103、GDX-105、GDX-203在最高耐受温度270 ℃下都可以实现二噁英的热脱附,证实了碳基吸附剂作为二噁英热吸附/脱附的吸附剂的可行性。310 ℃下,在丝光沸石上1,2,3,4-TCDD可实现热脱附,而1,2,3,8,9-PCDF在相同条件下无法实现热脱附,体现了沸石对二噁英同系物的选择性吸附的特性。而硅藻土和蒙脱土对气相中的二噁英几乎没有吸附能力,不适合作为二噁英热捕集的吸附剂。弗洛里硅土、硅胶、氧化铝、GDX-102、GDX-103和GDX-203对1,2,3,4-TCDD、1,2,3,8,9-PCDF都具有很强的吸附能力,因此被初步选为二噁英的候选吸附剂。通过比较120 ℃下和270 ℃下二噁英在这6种吸附剂上的ln*K*_SA_,发现弗洛里硅土在两个温度点下的保留体积都是最大的;在侧重低温下二噁英的热捕集性能时,弗洛里硅土是捕集二噁英的最佳吸附剂;而GDX-102是6种吸附剂中ln*K*_SA,270 ℃_最小的,在侧重高温下二噁英的热脱附性能时,GDX-102是二噁英热脱附的最佳吸附剂。同时,硅胶、GDX-103和GDX-203的ln*K*_SA,120 ℃_和ln*K*_SA,270 ℃_与GDX-102相近,也可以作为快速热吸附/脱附的材料。该文通过系统评价1,2,3,4-TCDD和1,2,3,7,8-PCDF在11种吸附剂的热吸附/脱附性能,对简化二噁英的采样和制备过程提供了新的解决思路,为实现二噁英的热捕集提供了技术支撑。

## Abstract (en)

The key to the online thermal capture of dioxins is the appropriate choice of adsorbent for efficient capture at low temperatures and rapid desorption at high temperatures. Efficient adsorbents can allow for capture and separation during the online monitoring of dioxins or during offline dioxin tests. In this study, 1,2,3,4-tetrachlorodibenzo-*p*-dioxin (1,2,3,4-TCDD) and pentachlorodibenzofuran (1,2,3,8,9-PCDF) were used as model compounds for dioxin monomers, and an electron capture detector (ECD) was used to detect the dioxin signals. The retention volumes of 1,2,3,4-TCDD and 1,2,3,8,9-PCDF on 11 types of adsorbents were determined using a packed-column gas chromatography system. Then, the corresponding van der Hoff equation was established, and these adsorbents were evaluated for the thermal trapping of dioxin. The linear coefficients of determination (*R*^2^) of these adsorbents were greater than 0.96, which indicated a strong correlation between the adsorption capacity and 1/*T* (*T*: temperature). The gas-solid partition coefficients (*K*_SA_) of the adsorbent at 120, 150 and 180 ℃ were predicted according to the van der Hoff equation, which was obtained in an earlier study. Among the 11 adsorbents, florite had the largest adsorption capacity at 120, 150, and 180 ℃, especially at 120 ℃; the *K*_SA_ values for 1,2,3,4-TCDD and 1,2,3,8,9-PCDF on florite were as high as 1.82×10^8^ m^3^/g and 1.46×10^13^ m^3^/g, respectively. Carbon-based adsorbents of the Chinese stilbene polymer porous microspheres GDX series, GDX-101, GDX-102, GDX-103, GDX-105, and GDX-203, can facilitate thermal desorption below 270 ℃, which is the maximum tolerance temperature for series 1 and 2, thus providing evidence for the feasibility of using these adsorbents for the thermal adsorption/desorption of dioxins. When the detection temperature is less than 310 ℃, 1,2,3,4-TCDD is thermally desorbed from mordenite, but 1,2,3,8,9-PCDF is not; this indicates the selective adsorption of dioxin monomers on zeolite. However, diatomite and montmorillonite have poor adsorption capacity for dioxins in the gas phase, thus being unsuitable for the thermal trapping of dioxins. Florite, silica gel, alumina, GDX-102, GDX-103, and GDX-203, which have strong adsorption capacities, were selected as possible absorbents for the next evaluation. Comparison of the ln*K*_SA_ values of dioxin monomers on the same adsorbent at 120 ℃ and 270 ℃ revealed that the retention volume of florite was the largest at both temperatures. When the thermal trapping performance of dioxin at low temperatures is considered, florite is thought to be the best among the 11 adsorbents for capturing dioxins. However, when the desorption performance at high temperatures is considered, GDX-102 is the best adsorbent for the thermal desorption of dioxins, and its ln*K*_SA,270 ℃_ is the smallest among those for the aforementioned six adsorbents. The ln*K*_SA,120 ℃_ and ln*K*_SA,270 ℃_ values of silica gel, GDX-103, and GDX-203 are similar to those of GDX-102, and hence, they can also be used as rapid thermal adsorption/desorption materials. In this study, the thermal adsorption/desorption properties of 1,2,3,4-TCDD and 1,2,3,7,8-PCDF on 11 adsorbents were systematically evaluated to obtain a new solution for the sampling and preparation of dioxins and to provide technical support for the thermal capture of dioxins. It should be noted that these results were obtained under ideal conditions of nitrogen, without considering the influence of the complex conditions of flue gas (such as moisture and CO_2_) on the thermal capture. To achieve the thermal capture of dioxins in incineration flue gas, it is necessary to carry out the relevant evaluation and test research in a flue gas atmosphere.

二噁英是一种受到高度关注的超痕量有毒有机污染物,主要产生于钢铁冶炼、垃圾焚烧等高温过程。高温源二噁英的采样需要收集颗粒相、气相和水相,因此后期需要复杂的样品制备过程,这也是实现二噁英在线监测的一个难点。筛选出一种可以热捕集烟气中二噁英的吸附材料,可以极大地简化二噁英采样和制备过程,对建立二噁英离线和在线检测系统均具有重要意义。

多孔碳材料因具有巨大的比表面积和与二噁英较强的相互作用力,成为广泛应用于水体和气体中二噁英的高效吸附剂^[1,2,3,4]^。目前,活性炭吸附已成为烟道气中二噁英减排的标准方法。多孔碳材料虽然对二噁英具有很强的吸附能力,但在高温下通常难以实现热脱附。Yang等^[5]^使用温度程序性脱附技术(TPD)测定了5种活性炭上的二噁英脱附温度,其结果均大于490 ℃。

非碳基吸附剂虽然对二噁英的吸附能力较弱,但由于易于实现热脱附而得到研究者的广泛关注。Dickson等^[6]^在研究二噁英的生成机理过程中,直接使用硅胶收集生成的二噁英。Yu等^[7]^发现纳米微孔活性硅在200 ℃时对烟道气中的二噁英脱除效率高达80%。Lasagni等^[8]^发现在200 ℃下加热2 h后,73%的二苯并-对-二噁英(DD)和79%的二苯并呋喃(DF)从硅胶中脱附。Guan等^[9]^使用热重实验发现二噁英在硅胶上的脱附温度约为200 ℃。Jager等^[10]^利用UTD-1 (0.75 nm×1.00 nm)、SSZ-24 (0.73 nm×0.73 nm)、ITQ (0.62 nm×0.72 nm) 3种不同孔径的沸石协同作用捕集气相中的二噁英,捕集效率达到了100%。Mercury等^[11,12]^考察了沸石的晶型、硅铝比、交换离子等因素对二噁英吸附容量的影响。Oliver等^[13]^发现沸石可选择性吸附四氯代和五氯代有毒同系物。Bullot等^[14]^报道了Beta型沸石及Na^+^-Beta型沸石对液相中2,3-二苯并-对-二噁英(2,3-DCDD)的吸附。Bullot等^[15]^以1,2-二氯苯和1,2,4-三氯苯作为二噁英的模型物,考察了金属有机框架MIL-101(Cr)对它们的吸附性能。Wang等^[16]^用密度泛函理论推断镍掺杂氮化硼纳米管是一种潜在的二噁英高效吸附剂。

本文的目的是筛选在低温下高效捕集并在一定的高温下可以快速完全脱附二噁英的吸附/脱附材料,从而简化二噁英的采样和制备过程,为未来二噁英在线检测系统的搭建提供技术支撑。本文考察了5种碳基的高分子多孔微球(1系列GDX-101、GDX-102、GDX-103、GDX-105和2系列GDX-203)和6种非碳基吸附剂(硅胶、氧化铝、弗洛里硅土、硅藻土、蒙脱土和丝光沸石)对两种二噁英单体1,2,3,4-四氯代二苯并-对-二噁英(1,2,3,4-TCDD)和1,2,3,8,9-五氯代二苯并呋喃(1,2,3,8,9-PCDF)的热吸附性能,根据范特霍夫方程预测了120、150、180 ℃ 3个温度点下的气固分配系数(*K*_SA_),通过比较120、270 ℃下的ln*K*_SA_,筛选出最佳的吸附材料。

## 1 实验部分

### 1.1 实验原理

气固分配系数是吸附质在气固两相中的平衡常数^[17]^,它与相对保留体积的关系见式(1)。


(1) *K*_SA_=*V*_G_/*M*

式中,*K*_SA_为气固分配系数,*V*_G_为相对保留体积(mL),*M*为固定相的质量(g)。

本实验中相对保留体积*V*_G_的计算见式(2)。


(2) *V*_G_=*V*_R_-*V*_M_=*v*(*t*_R_-*t*_M_)

式中,*V*_R_为保留体积,*V*_M_为死体积,*v*为载气流速,*t*_R_为保留时间,*t*_M_为死时间,*t*_M_通过测定二氯甲烷的出峰时间确定。

根据范特霍夫方程^[18]^, *K*_SA_与温度的关系见式(3)。


(3) ln*K*_SA_=-Δ*H/RT*+*C*

式中,Δ*H*为焓变,*R*为气体常数,*T*为温度,*C*为常数。

### 1.2 仪器和试剂

填充柱气相色谱系统Hewlett Packard 5890 series Ⅱ(带电子捕获检测器(ECD),美国Hewlett-Packard公司);玻璃管填充柱(200 mm×6.0 mm, 1.0 mm,艾特石英制品公司); 10 μL微量进样针(上海阿拉丁公司)。

1,2,3,4-TCDD、1,2,3,8,9-PCDF的质量浓度均为50.0±0.5 μg/mL(美国剑桥同位素实验室);石英砂(40~80目)(天津科密欧公司);氮气(99.9%)(大连永达气体站)。

碳基吸附剂:GDX-101、GDX-102、GDX-103、GDX-105和GDX-203均为40~60目(上海阿拉丁公司);非碳基吸附剂:高纯硅胶(35~60目)(美国Sigma公司)、丝光沸石(80~100目)(江苏先丰纳米材料科技有限公司)、活性氧化铝(40~60目) 、蒙脱土(80~100目) 、弗洛里硅土(40~60目) (上海阿拉丁公司)、硅藻土(40~60目) (天津科密欧公司)。

### 1.3 实验条件

1.3.1 色谱填料预处理

石英砂在马弗炉中500 ℃过夜,非碳基吸附剂在290 ℃、氮气保护下加热6 h,碳基吸附剂GDX系列在250 ℃、氮气保护下加热12 h。

1.3.2 实验操作步骤

用石英棉将玻璃管的底部塞紧,装入1 g石英砂,摇晃使石英砂平实,装入吸附剂,再装入1 g石英砂,用石英棉塞紧顶部。利用具有填充柱进样口的气相色谱仪完成热吸附/脱附试验。玻璃管前端接填充柱进样口,后端通过毛细柱转接ECD,实验流程图见图1。柱箱温度为恒温模式。在柱箱温度和ECD信号稳定后,用微量进样针依次注入50 μg/mL 1,2,3,4-TCDD溶液1 μL、50 μg/mL 1,2,3,8,9-PCDF溶液1 μL,记录二噁英出峰时间和实验温度。

**图 1:**
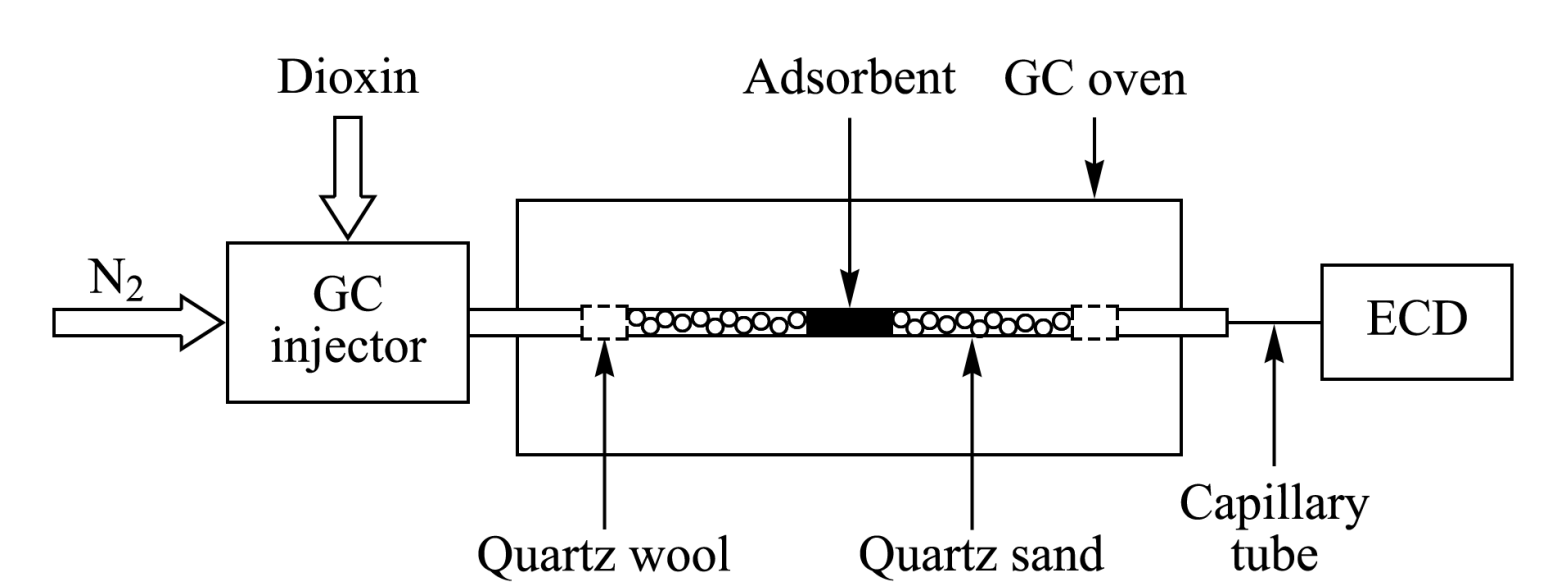
实验流程图

### 1.4 分析条件

进样室温度设为280 ℃;不分流进样;ECD温度设为310 ℃;非碳基吸附剂的柱箱温度上限为310 ℃,柱箱温度根据实验情况调整;GDX-101、GDX-102、GDX-103、GDX-105和GDX-203的最高耐受温度为270 ℃,柱箱温度分别设为250、255、260、265、270 ℃;氮气为载气;使用泡沫流量计测流速,每次测量至少为3次,取平均值,非碳基吸附剂的氮气流速为18 mL/min,碳基吸附剂的氮气流速为22 mL/min。

## 2 结果与讨论

### 2.1 高温条件下二噁英单体在不同吸附剂上的吸附性能

本文评价了11种不同吸附剂对1,2,3,4-TCDD、1,2,3,8,9-PCDF的吸附性能,实验结果见表1。

**表1:** 1,2,3,4-TCDD和1,2,3,8,9-PCDF在11种吸附剂上的吸附结果汇总

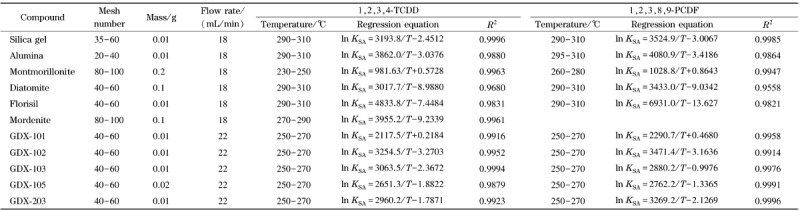

*K*_sA_: gas-solid partition coefficients; *T*: temperature.

表1中,各种吸附剂的范特霍夫方程的决定系数(*R*^2^)均大于0.96,表明本实验得出的ln*K*_SA_和1/*T*具有极强的线性相关性。相同条件下,1,2,3,8,9-PCDF的出峰时间都晚于1,2,3,4-TCDD,这是因为1,2,3,8,9-PCDF的相对分子质量比1,2,3,4-TCDD大,与吸附剂之间的相互作用力更强。在丝光沸石上,柱箱温度为270、275、280、285、290 ℃时,1,2,3,4-TCDD均有峰检出,但未检到1,2,3,8,9-PCDF峰,可能的原因是沸石对二噁英的吸附作用主要来源于沸石孔道对二噁英的限位作用^[10,19]^。当柱箱温度范围为290~310 ℃时,高纯硅胶、氧化铝和弗洛里硅土上,1,2,3,4-TCDD、1,2,3,8,9-PCDF的出峰时间均在30 min内,其中,1,2,3,4-TCDD、1,2,3,8,9-PCDF在弗洛里硅土上的线性回归方程的斜率在11种吸附剂中均为最大值。在蒙脱土上,柱箱温度为230~250 ℃时,1,2,3,4-TCDD在30 min内出峰;柱箱温度为260~280 ℃时,1,2,3,8,9-PCDF在30 min内出峰。在表1中,蒙脱土的范特霍夫方程的斜率最小,表明蒙脱土对二噁英的吸附能力较差。GDX的1系列和2系列是二乙烯苯和苯乙烯等高聚物构成的高聚物多孔小球,最高耐受温度为270 ℃,因此GDX系列的实验温度都小于等于270 ℃。在5种GDX吸附剂上,1,2,3,4-TCDD, 1,2,3,8,9-PCDF都可以在270 ℃以下时实现热脱附,并且结构保持完整,证实了二噁英单体在多孔高聚物小球上热脱附的可行性。

### 2.2 低温条件下二噁英单体在不同吸附剂上的气固分配系数估算

烟道气中二噁英采样温度通常在120~130 ℃,本文依据范特霍夫方程的变形式(式(4)),计算出120、150、180 ℃ 3个温度点下不同吸附剂对两种二噁英单体的*K*_SA_,具体数据见表2。


(4) *K*_SA_=e^-Δ^*^H^*^/^*^RT^*^+^*^C^*

**表 2:** 1,2,3,4-TCDD和1,2,3,8,9-PCDF在不同吸附剂上120、150、180 ℃时的气固分配系数

Compound	1,2,3,4-TCDD				1,2,3,8,9-PCDF			
	120 ℃	150 ℃	180 ℃		120 ℃		150 ℃	180 ℃
Silica gel	31203.6	152.2	4.4		282647.6		794.0	15.8
Alumina	4548112.1	7284.9	99.7		19257081.9		21415.9	229.9
Montmorillonite	0.006	0.001	0.0004		0.016		0.002	0.0007
Diatomite	10.4	0.07	0.002		316.9		1.0	0.02
Florisil	1.8×10^8^	57608.4	267.9		1.5×10^13^		1.4×10^8^	63770.4
Mordenite	20142.5	27.6	0.3					
GDX-101	57.3	1.7	0.2		311.6		6.8	0.5
GDX-102	22811.6	100.5	2.7		154696.9		475.1	10.0
GDX-103	11458.1	69.4	2.3		12008.4		98.8	4.0
GDX-105	599.7	7.2	0.4		2608.0		26.1	1.2
GDX-203	8653.5	62.3	2.3		80896.3		348.0	9.2

如表2所示,120 ℃时,1,2,3,4-TCDD在不同吸附剂上的*K*_SA_从大到小依次为:弗洛里硅土>氧化铝>硅胶>GDX-102>丝光沸石>GDX-103>GDX-203>GDX-105>GDX-101>硅藻土>蒙脱土。150 ℃时,1,2,3,4-TCDD在不同吸附剂上的*K*_SA_从大到小依次为:弗洛里硅土>氧化铝>硅胶>GDX-102>GDX-103>GDX-203>丝光沸石>GDX-105>GDX-101>硅藻土>蒙脱土。180 ℃时,1,2,3,4-TCDD在不同吸附剂上的*K*_SA_从大到小依次为:弗洛里硅土>氧化铝>硅胶>GDX-102>GDX-203>GDX-103 >GDX-105 >丝光沸石>GDX-101>硅藻土>蒙脱土。而120、150、180 ℃时,1,2,3,8,9-PCDF在不同吸附剂上的*K*_SA_从大到小均表现为:弗洛里硅土>氧化铝>硅胶>GDX-102>GDX-203>GDX-103 >GDX-105> GDX-101>硅藻土>蒙脱土。

温度为120、150、180 ℃时,1,2,3,8,9-PCDF在同一吸附剂的气固分配系数都大于1,2,3,4-TCDD,这与高温下相同温度时1,2,3,8,9-PCDF的保留体积都大于1,2,3,4-TCDD的表现一致。温度为120、150、180 ℃时,1,2,3,4-TCDD、1,2,3,8,9-PCDF在弗洛里硅土上的*K*_SA_都为最高,特别是在120 ℃下,1,2,3,4-TCDD、1,2,3,8,9-PCDF在弗洛里硅土上的*K*_SA_分别高达1.82×10^8^ m^3^/g、1.46×10^13^ m^3^/g。而温度为120、150、180 ℃时,在蒙脱土上的*K*_SA_都为最低。

综合表2的1,2,3,4-TCDD和1,2,3,8,9-PCDF在120 ℃时不同吸附剂上的*K*_SA_,硅胶、氧化铝、弗洛里硅土、GDX-102、GDX-103和GDX-203均有较强的吸附保留能力,可以作为候选的120 ℃二噁英采样的热吸附材料。

### 2.3 高低温气固分配系数的比较和吸附剂的选择

本文选取120 ℃作为二噁英的吸附温度,270 ℃作为二噁英的脱附温度,计算相应的ln*K*_SA,120 ℃_和ln*K*_SA,270 ℃_。ln*K*_SA,120 ℃_代表了120 ℃下吸附剂的保留能力,值越大表示吸附能力越强,越适于低温下二噁英采样;ln*K*_SA,270 ℃_代表了270 ℃下吸附剂的保留能力,值越小,表示越容易发生热脱附。因此,ln*K*_SA,120 ℃_/ln*K*_SA,270 ℃_的值越大,表示低温吸附越强或高温脱附越容易,反之,表示低温吸附越弱或高温脱附越难。

如图2所示,6种吸附剂对1,2,3,4-TCDD的ln*K*_SA,120 ℃_/ln*K*_SA,270 ℃_从大到小依次为:弗洛里硅土(3.14)>GDX-102(2.71)>氧化铝(2.59)>GDX-103(2.58)=硅胶(2.58)>GDX-203(2.49)。弗洛里硅土在120 ℃下的ln*K*_SA_(32.83)最大,并且ln*K*_SA,120 ℃_/ln*K*_SA,270 ℃_在6种吸附剂中为最大,吸附和脱附效果最明显,但在270 ℃下的ln*K*_SA_(10.45)仅次于氧化铝(11.27),在高温下对1,2,3,4-TCDD的保留能力仍然较强,在优先考虑低温下对二噁英的吸附能力条件下,弗洛里硅土是最佳的吸附剂,并且可以通过进一步提高弗洛里硅土的脱附温度来改善1,2,3,4-TCDD的热脱附效果。120 ℃下,硅胶、GDX-102、GDX-103和GDX-203对1,2,3,4-TCDD的ln*K*_SA_相近,但270 ℃下GDX-102的ln*K*_SA_在6种吸附剂中为最小,并且ln*K*_SA,120 ℃_/ln*K*_SA,270 ℃_=2.72仅次于弗洛里硅土。在侧重1,2,3,4-TCDD的快速完全热脱附的条件下,GDX-102是最佳的吸附剂,且由于GDX系列是高分子聚合物,因此对有机污染物具有更强的选择性,然而受限于GDX-102的最高耐受温度,GDX-102无法通过进一步升温来改善脱附效果。

**图 2:**
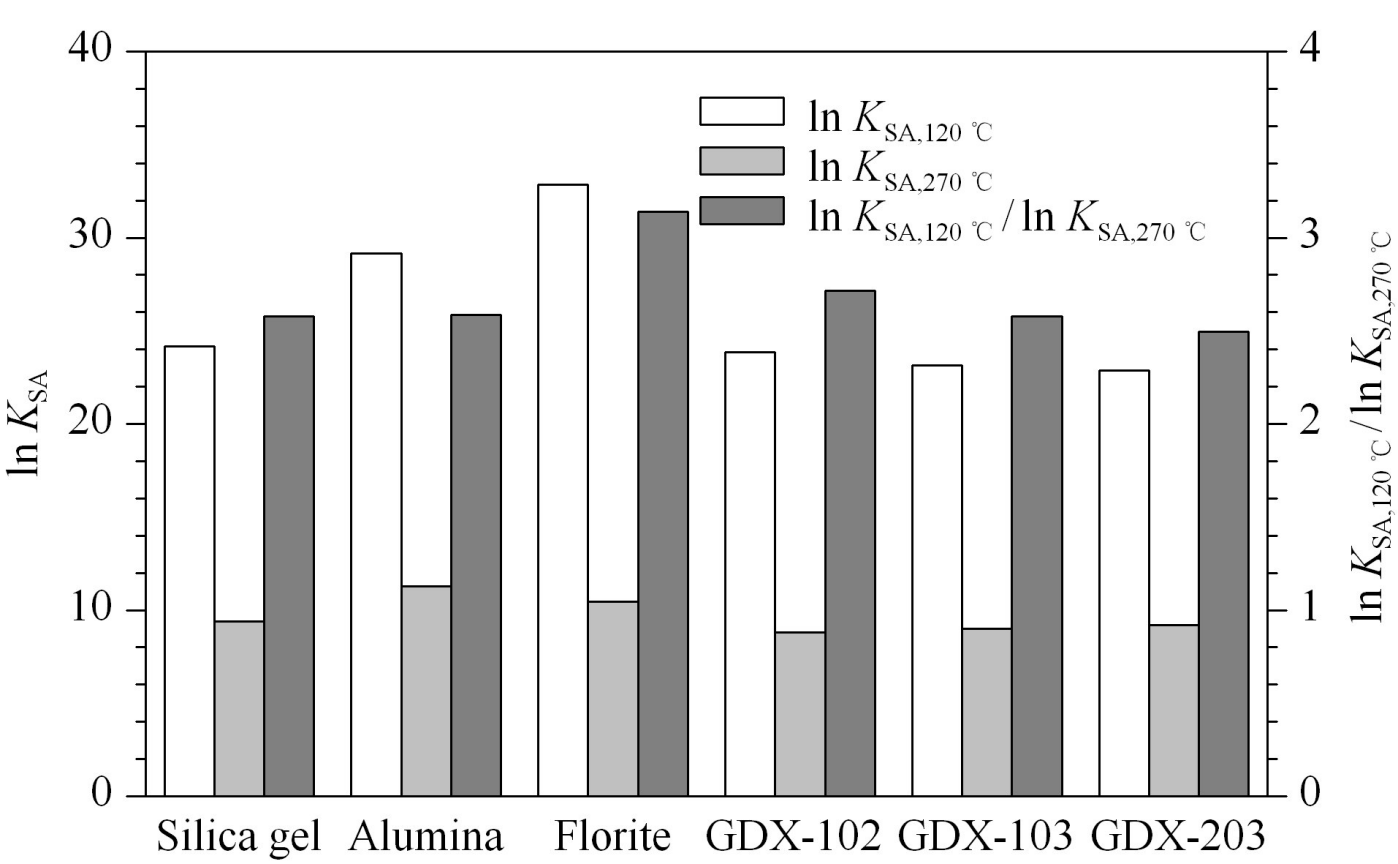
1,2,3,4-TCDD在不同吸附剂上在120、270 ℃时的ln*K*_SA_和ln*K*_SA,120 ℃_/ln*K*_SA,270 ℃_

如图3所示,6种吸附剂上1,2,3,8,9-PCDF的ln*K*_SA,120 ℃_/ln*K*_SA,270 ℃_从大到小依次为弗洛里硅土(3.66)>GDX-102(2.66)>硅胶(2.62)>氧化铝(2.61)> GDX-203(2.52)>GDX-103(2.35)。

**图 3:**
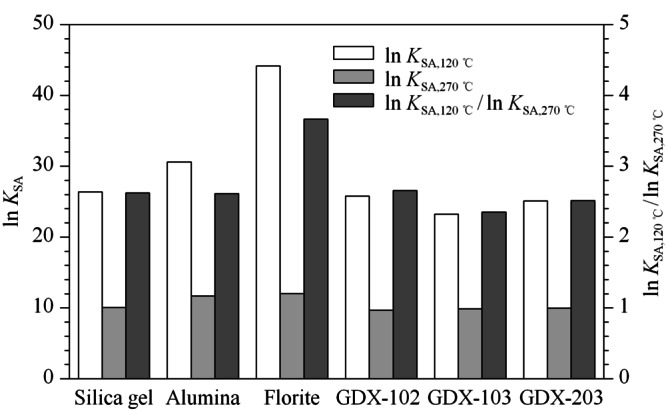
1,2,3,8,9-PCDF在不同吸附剂上在120、270 ℃时的ln*K*_SA_和ln*K*_SA,120 ℃_/ln*K*_SA,270 ℃_

弗洛里硅土在120 ℃下的ln*K*_SA_(44.13)最大,并且ln*K*_SA,120 ℃_/ln*K*_SA,270 ℃_在6种吸附剂中为最大,吸脱附效果最明显,但在270 ℃下的ln*K*_SA_(10.45)在6种吸附剂中为最大,在高温下对1,2,3,8,9-PCDF的保留能力仍然较强,在优先考虑低温下对1,2,3,8,9-PCDF的吸附能力条件下,弗洛里硅土仍是最佳的吸附剂。120 ℃下,硅胶、GDX-102、GDX-103和GDX-203对1,2,3,8,9-PCDF的ln*K*_SA_相近,270 ℃下GDX-102的ln*K*_SA_在6种吸附剂中仍为最小,并且ln*K*_SA,120 ℃_/ln*K*_SA,270 ℃_=2.65仅次于弗洛里硅土,在侧重1,2,3,8,9-PCDF的高温下快速完全热脱附的条件下,GDX-102是最佳的吸附剂。

综上,在侧重二噁英在低温下的吸附效果时,弗洛里硅土是最佳的吸附剂。弗洛里硅土在高温下仍然对二噁英具有较强的吸附能力,但可以通过进一步提高脱附温度来改善二噁英热脱附性能。在侧重二噁英在高温下(270 ℃)下的脱附效果时,GDX-102是最佳的吸附剂,同时,硅胶、GDX-103和GDX-203的ln*K*_SA,120 ℃_/ln*K*_SA,270 ℃_与GDX-102相近,在一定条件下也可以取代GDX-102作为二噁英捕集和快速热脱附的材料。

## 3 结论

本文比较了1,2,3,4-TCDD和1,2,3,8,9-PCDF在11种吸附剂上的吸附性能,得到了11种吸附剂上的1,2,3,4-TCDD的范特霍夫方程和10种吸附剂上的1,2,3,8,9-PCDF的范特霍夫方程,其决定系数*R*^2^都高于0.96。根据范特霍夫方程的估算,在11种吸附剂中,弗洛里硅土表现出对1,2,3,4-TCDD、1,2,3,8,9-PCDF最强的吸附能力,特别是在120 ℃时,1,2,3,4-TCDD、1,2,3,8,9-PCDF在弗洛里硅土上的*K*_SA_分别高达1.82×10^8^ m^3^/g、1.46×10^13^ m^3^/g。在侧重二噁英在低温下的吸附能力时,弗洛里硅土是最佳的吸附剂,在侧重二噁英在高温下的脱附能力时,GDX-102是最佳的吸附剂。

本工作筛选出弗洛里硅土、GDX-102、氧化铝、GDX-103、硅胶和GDX-203等6种对二噁英具有较强吸附/脱附性能的吸附剂,为二噁英在线检测系统的搭建提供了技术支撑。然而,需要指出的是,上述结果均是在氮气保护的理想条件下获得的,没有考虑烟气的复杂条件(如水分、二氧化碳、氮氧化物和其他有机污染物如苯、氯苯、氯酚等)对热捕集的影响,真正实现焚烧烟气二噁英的热捕集还需要进一步开展烟气氛围下的相关评价与测试研究。

## References

[1] Zhou X, Li X, Xu S, et al. Environ Sci Pollut Res, 2015,22:10463 10.1007/s11356-015-4180-9 25728198

[2] Cerasa M, Benedetti P, Stefanis A D, et al. Chemosphere, 2020,239:124666 31479911 10.1016/j.chemosphere.2019.124666

[3] Guo Y Y, Li Y R, Zhu T Y, et al. Chem Eng J, 2016,283:1210

[4] Long R Q, Yang R T. J Am Chem Soc, 2001,123(9):2058 11456830 10.1021/ja003830l

[5] Yang R T, Long R Q, Padin J, et al. Ind Eng Chem Res, 1999,38(7):2726

[6] Dickson L C, Lenoir D, Hutzinger O. Environ Sci Technol, 1992,26(9):1822

[7] Yu Y, Ling Y, Li X, et al. Baosteel Technology, 2016,5:18

[8] Lasagni M, Collina E, Tettamanti M, et al. Environ Sci Technol, 1996,30(6):1896

[9] Guan Y, Liu Y, Wu W, et al. Langmuir, 2005,21(9):3877 15835949 10.1021/la0468545

[10] Jager R, Schneider M A, Behrens P, et al. Chem-Eur J, 2011,10(1):247

[11] Mercury M, Denoyel R, Simon-Masseron A, et al. Adsorption, 2011,17(4):747

[12] Mercury M, Zouaoui N, Simon-Masseron A, et al. Micropor Mesopor Mat, 2013,177:25

[13] Oliver S, Laurence T, Angelique S, et al. Chemosphere, 2020,259:127457

[14] Bullot L, Abda M B, Simon-Masseron A, et al. Adsorption, 2016,23(1):1

[15] Bullot L, Vieira-Sella L, Chaplais G, et al. Environ Sci Pollut R, 2017,23(1):1 10.1007/s11356-017-0242-5 28952020

[16] Wang R, Zhang D, Liu C. Chemosphere, 2017,168:18 27776234 10.1016/j.chemosphere.2016.10.050

[17] Goss K U, Buschmann J, Schwarzenbach R P. Environ Sci Technol, 2004,38(13):3667 15296319 10.1021/es035388n

[18] Zhang H, Zhao S, Yu Y, et al. Environ Sci Technol, 2010,44(10):3677 20402500 10.1021/es9034705

[19] Ben-Abda M, Scha F O, Zerega Y. Micropor Mesopor Mat, 2015,217:178

